# Digital pathology implementation in cancer diagnostics: towards informed decision-making

**DOI:** 10.3389/fdgth.2024.1358305

**Published:** 2024-05-30

**Authors:** Oksana Sulaieva, Oleksandr Dudin, Olena Koshyk, Mariia Panko, Nazarii Kobyliak

**Affiliations:** ^1^ Medical Laboratory CSD, Kyiv, Ukraine; ^2^ Endocrinology Department, Bogomolets National Medical University, Kyiv, Ukraine

**Keywords:** digital pathology, Augmented Human Intelligence, informed consent, physician-patient relationship, bioethics

## Abstract

Digital pathology (DP) has become a part of the cancer healthcare system, creating additional value for cancer patients. DP implementation in clinical practice provides plenty of benefits but also harbors hidden ethical challenges affecting physician-patient relationships. This paper addresses the ethical obligation to transform the physician-patient relationship for informed and responsible decision-making when using artificial intelligence (AI)-based tools for cancer diagnostics. DP application allows to improve the performance of the Human-AI Team shifting focus from AI challenges towards the Augmented Human Intelligence (AHI) benefits. AHI enhances analytical sensitivity and empowers pathologists to deliver accurate diagnoses and assess predictive biomarkers for further personalized treatment of cancer patients. At the same time, patients’ right to know about using AI tools, their accuracy, strengths and limitations, measures for privacy protection, acceptance of privacy concerns and legal protection defines the duty of physicians to provide the relevant information about AHI-based solutions to patients and the community for building transparency, understanding and trust, respecting patients' autonomy and empowering informed decision-making in oncology.

## 1 Introduction

Pathology is essential in cancer diagnostics and guides further clinical decisions and patient management (1). The complexities and multimodality of cancer at the individual level complicate the relevant assessment of clinical and laboratory data, demographic and anthropometric features, lifestyle, personal and family history, tumor stage, histological and molecular type of tumor, its heterogeneity and evolution during the treatment during clinical decision-making. From this perspective, artificial intelligence (AI) was recognized to play an essential role in supporting health system management, especially in cancer care where AI-based solutions facilitate the assessment of public health threats, improve diagnostic access, shorten turnaround time and enhance the accuracy in cancerous lesions detection (2). Electronic health records, technological advancements and AI-based tools allow to minimize the existing constraints fostering accurate patient-specific data analysis and enabling precision medicine approaches for individualizing cancer care and improving patient outcomes (3, 4). Implementation of precision medicine and technological advances in pathology and genetics defined the shift from physical histological slide microscopy toward digital format facilitating digital pathology (DP) evolution (4).

The primary canon of DP is rooted in obtaining high-resolution whole slide images (WSI) for further observation, sharing, storage, and advanced analysis. Digital scanner implementation in the workflow has determined the development of the next step toward pathology laboratory automation and digitalization by addressing artificial intelligence (AI)-based approaches for WSI recognition of particular patterns for diagnostic, prognostic and prediction purposes (5). AI, Internet to Things (IoT) concept and emerging technologies transformed the routine pathology practice enabling assessment of WSI, their sharing for “second opinion” consultations, reducing human and infrastructural resource burden (6) and mining of multiple visual features for more accurate diagnostics, prognosis, prediction of response to various treatments and better patient outcomes (7). With the high-speed progress in cancer multi-omics studies, AI-based solutions have been facilitating the discovery of novel biomarkers and precision medicine implementation (1). AI-based tools in pathology were also essential for developing molecular classification of various cancers (TCGA project) and discovering novel approaches for predicting genetic alterations, and drug discovery for better treatment outcomes (7). Thus, the application of DP in convergence with AI-based tools enabling WSI analysis for diagnostic and prognostic purposes provides multiple benefits for both healthcare professionals and patients by empowering pathologists to deliver accurate diagnoses and assess predictive biomarkers for more personalized treatment (4). Although recent policy strategies in many developed countries directed funding to support the deployment of digital pathology, there are still gaps in the legal dimension and ethical concerns about ensuring patients' autonomy, protection and fair access to novel technologies (8).

The digital transformation of pathology impacts physician-patient relationships. The ethical dimensions of the physician-patient relationships are shaped by Beauchamp and Childress' bioethical principles and respect for autonomy, beneficence, non-maleficence, and justice (9). A growing body of studies addresses questions of the safety and quality of AI-based tools in relation to physicians' duties to promote patients' best interests (aligning with beneficence) and minimizing harm (according to non-maleficence imperative) (10, 11). Challenges related to physician use of AI-based tools include automation bias, technical limitations, data governance and resource allocation, fair access to novel technologies and transparency related to patients' autonomy and further decision-making (12–14). Many ethical concerns about patient autonomy when using AI have been identified, including disclosure and informed consent process, privacy and security issues, which raise the need for transparency and building trust in AI-powered algorithm applications (4, 10). From this perspective, patient autonomy is often beyond the scope of ethical analysis because the incorporation of AI algorithms has changed not only workflow but also the essence of the diagnostic process in pathology laboratories. Only a few studies address the patient-centric approach to implementing AI-based tools in pathology (15). AI has been becoming an additional “participant” in diagnostic and therapeutic pathways affecting physician-patient relationships, accessing patient data and providing recommendations concerning sensitivity to various treatments. Implementing DP affects the patient's right to know about diagnostic and treatment pathways. Questions arise about whether patients be informed about the use of AI for diagnostics, what information should they know, and if patients should have a choice between traditional pathology and DP-supported diagnostics. How these questions are answered have an impact on patients' rights, protection, and autonomy.

This paper argues that physicians using digital and computational pathology tools for cancer diagnostics have an ethical obligation to transform the physician-patient relationship for enable informed and responsible decisions.

## 2 Ethical imperative for transparency of using DP tools

The importance of the transparency of using AI-supported solutions in pathology as they affect clinical decisions has reached a global convergence (15). Most biases concerning AI are related to low literacy and unfounded fears, so transparency can foster trust and define the responsible and relevant use of the innovation. Trust in novel technologies relies on proficiency and rises along with the experience. For instance, the Swedish report on using WSI in 2006–2017 demonstrated that only 38% of cases were diagnosed digitally, while in the rest of the cases, pathologists switched between digital and glass slides, reflecting the lack of confidence when signing out reports with DP (16, 17). However, in 2017, some studies demonstrated >90% acceptance of WSI by pathologists for diagnostic issues (18). Moreover, driven by the COVID-19 pandemic transition toward virtual activities fostered pathologists to network education and consultations using and sharing WSI. This defined an acceptance of virtual meetings and slides as a substitute for glass specimens for regular practice (19). Similarly, the first implementation of AI-based computational pathology tools for image analysis relied on explainability and causative approach (20). In 1995 FDA approved the first AI-supported medical device for supporting cytologists in recognizing abnormal cells in PAP smears, and since that time, AI-driven solutions have been playing a pivotal role in cervical screening. In 2023, ML algorithms was reported to outperform the standard clinical risk model for assessing 5-year risk of breast cancer development, underscoring AI's role in cancer management (21).

Nevertheless, transparency is essential for DP tool developers, policymakers, technology users (pathologists and clinicians), and patients whose data are handled by AI-powered solutions. Guarantee that AI-based solutions are transparent, unbiased, and ethically justifiable is paramount to maintaining the trust of various stakeholders (4). The depth of transparency at least partly relies on the type of machine learning (ML) methods applied (supervised, unsupervised or reinforced learning) (22). A choice among a wide range of algorithms within various ML platforms depends on the goal and tasks, the type and number of data to be analyzed and the size of the dataset, the learning approach, the level of accuracy, the need for clustering, hierarchical output or speed in data assessment, etc. Many ML algorithms do not reveal the principles and logic of the underlying decisions (23, 24). The “black box” paradox defines the obscure nature and the non-explainability of the unsupervised AI-based decisions that hinder the trust and stumble the widespread integrations of computational pathology tools in diagnostic settings. These challenges drove the development of an explainable AI (XAI) approach for enhancing the interpretability, explainability, justifiability, contestability and transparency of the applied AI-models (25).

Despite the exciting results of AI-based solutions development and testing under controlled conditions, the real-world application demonstrates discrepancies from the initial discoveries. The causes are rooted in various reasons including the training set variety and size, depth of data incorporated in the analysis, the dependence on technical parameters (scanner, processing, staining), quality of primary data annotation, etc. (26). Despite the impressive results, ML/AI applications can be undermined by common technical factors, including blur, tissue folds, tears, or color variations. This defined the need for normalization algorithms and color augmentation as well as the requirement to histopathology professionals to revalidate AI-based applications aligned with the preanalytical stage of workflow updates. A possible “replication crisis” in DP and the clinical harm of using unreliable AI-based solutions in practice dictated the need for proper oversight (27, 28).

To attain greater transparency, institutions that develop or deploy AI-based systems should enhance the disclosure of information, about AI-application benefits confirmed by real-world data and healthcare providers' experience (10). The following issues are typically discussed with stakeholders: use of AI (29), source code and data use, evidence about AI tools` performance and their limitations (30), legal regulations and oversight (10), data protection strategies (31), as well as communication with the community for building trust (10). Besides, SPIRIT-AI and CONSORT-AI guidelines for clinical trials using AI addressed the existing challenges and were extended to include additional requirements to clearly describe the intended use of the AI, indications for how to use the AI intervention in the clinical setting, details on the data inputs to train the AI tool, and the outputs it produces, descriptions of the identification and revision of the errors, as well as human-computer interplay (28). Thus, transparency is the prerequisite for the responsible development and implementation of computational pathology in practice.

## 3 Ethical requirements for privacy protection when using DP tools

Despite the Ethically Aligned Designed initiative led by the Institute of Electrical and Electronics Engineers (IEEE) for governing and standardizing AI-based technologies (32), and Digital Pathology Association activities to guide AI-based pathology practice ethically, gaps in DP ethical status and transparency to patients persist. Good Clinical Laboratory Practice (GCLP), ISO 15189 and Clinical Laboratory Improvement Amendments (CLIA) regulations dictate the need to ensure the ethical integrity of all procedures related to data acquisition and management in laboratories. WSI analysis falls under the specifications of these regulations (33). Patients must provide informed consent for their data use (including digital slides and associated personal information) therefore they must be informed about DP application (33, 34).

Using DP tools for sharing slides during the “second opinion” consultations addressing other experts' opinions when diagnosing cases of high complexity, also requires privacy protection following the federal Health Insurance Portability and Accountability Act (HIPAA) or the European General Data Protection Regulations (GDPR), which safeguards data protection for individuals within the USA or European Union (EU) respectively (35, 36). The EU's GDRP Regulation and OECD Artificial Intelligence Papers have already articulated the policies for increasing transparency of using AI in the Health sector. The most important recommendations include (1) public and provider engagement in discussing opportunities, risks, and concerns; (2) transparent reporting for AI performance and AI incidents, including impacts, lessons learned, and adjustments; (3) establishing rules about data control and (4) incentivizing and overseeing adherence to responsible AI practices and codes of conduct (2). Besides, the recently updated EU AI Act (37), also addresses the obligation for transparency for providers and users of AI. Although the strict limitations and overseeing health data sharing is justifiable from the subjects' protection perspective, these also restrain research in development of life-saving treatments and personalized medicine. Coping with this intrinsic conflict, the European Commission proposed the regulation to establish the European Health Data Space. The Commission articulated rules focused on two main goals: (1) to put citizens at the center of healthcare, giving them full control over their data to obtain better healthcare across the EU; (2) to open up data for research and public health (38).

Despite the regulations on protecting personal data and ensuring confidentiality in healthcare, the privacy risks related to WSI storage and sharing have not been completely clarified. WSI belongs to a specific data category, as a digital slide is just an image of high resolution in gigapixels or tens of gigapixels reflecting the detailed structure of tissue samples obtained, processed, cut, stained, and scanned in the laboratory. From this perspective, WSI could be categorized as low-risk data (39). However, the scan of the histological specimen can be labeled with a patient identifier and also linked to clinical or laboratory data, so WSI should be considered as sensitive as other clinical data. These factors justify the guidelines for releasing WSI as personal data that requires regulating data transfer and/or processing legally, pseudonymizing and minimizing the data set, safeguarding data via technical and organizational measures and considering information leakage from AI models (40). Thus, digital pathology systems that handle patient data associated with WSI should align with regulations to safeguard personal information when storing, transmitting, and allowing access to third parties, which requires the corresponding security measures to protect the integrity of patient data. However, there are still no articulated requirements for the list of data to be collected for proper analysis with respect to the clinical context of an individual`s case, characteristics of data storage capacity and the regulations for the WSI sharing with respect to the standardized annotations, etc. Lack of common legal and operational standards with respect to data input and protection, computational pathology algorithms will vary in quality, timeliness, cost, and unclear outcomes. Thus, standardization of computational pathology solutions development, validation and use will simplify transparency and trustworthiness of responsible AI-application.

On the other hand, GDPR requires that data subjects have a right to “meaningful information about the logic involved” in data analysis (15). This requires the disclosure of not only using AI-based tools but also some clarification about how AI decision-making works (41). The evolving nature of AI defines the continuous learning and emerging new properties that define both benefits (for enhancing diagnostic accuracy and widening predictive potential) and risks (for instance errors, and discrepancies in case of rare feature recognition) that require the proper risk assessment, post-implementation monitoring, overseeing and corresponding disclosure to patients, clinicians and community. The US Food and Drug Administration (FDA) and *In Vitro* Diagnostic Medical Device Directive (CE IVD) approved several algorithms for some predictive biomarkers assessment (such as ER, PR, HER-2 and Ki-67 expression in breast cancer, Glisson grading in prostate cancer, etc.) (42), regulating the clinical utility of DP. At the same time, the FDA proposes a regulatory amendment for AI/ML-based software to be considered as a medical device, transforming the perception of AI (FDA) (43). Similarly, in the European Union (EU), any AI-based devices and software declared to be used for diagnostics, disease prevention, monitoring and treatment are considered medical devices (44). To guide the use of software in clinical practice the FDA also proposed Good Machine Learning Practices (GMLPs), providing 10 basic principles to promote the safe and effective application of “medical devices that use artificial intelligence and machine learning (AI/ML)” (43). At the same time, GMLPs also address the Performance of the Human-AI Team. Such an approach drives the transformation of users' perception from Artificial intelligence challenges in the Augmented Human Intelligence (AHI) paradigm.

## 4 From artificial to Augmented Human Intelligence

A trend to move from the “technology-centric” approach toward humanization of using ML-based tools in pathology, mirrored in the concept of AHI has occurred. While some AI- algorithms are independent and operate autonomously, AHI uses ML to enhance human intelligence by providing actionable data. AHI efficiency relies on the synergy between human experience and ML ability to learn facilitating continuous improvement without additional programming. The transformation of DP core concept from AI toward AHI is based on the realistic assessment of the procedures and responsibilities during the diagnostic process in pathology (45). AHI relies on the use of computers and AI-based algorithms to facilitate and accelerate pathologists' role in image and data analysis (13). For example, the use of convoluted neural networks allows for the decomposition of a slide image and extraction of visual and subvisual features, typical for particular histological and molecular subtypes of tumors, which can be easily overlooked during human-based microscopy (46). Optimized AI-based biomarker assessment can save time and provide pathologists with accurate quantification of various protein expressions, increasing analytical sensitivity and helping to define high-risk patients and pre-select individuals for different therapies in line with the patients' best interests (47). Thus, AI application provides additional values for histopathological assessment.

AI-powered algorithms, however, do not provide uncontrolled autonomous decisions for their specific application. Fears that AI can replace pathologists and/or physicians are unsubstantiated. The AHI's central question is how AI can support and improve pathologists' work, providing additional benefits for precise diagnostics and predicting patient responses to therapy. Overall, AHI promises to enhance diagnostic accuracy in pathology and the performance of healthcare (45). Human-AI-teams seem to be more efficient in adjusting to rapidly changing treatment guidelines in oncology which incorporate various novel biomarkers and therapeutic agents. However, AHI applications should rely on verified validated models demonstrating high performance and reproducibility of not only machine learning algorithms alone but also AI-pathologists interplay. In this context, having “human in the loop,” addresses the need to assess the performance of the Human-AI team to consider the model`s output interpretability and the responsibility of human experts (15). Clear roles of AI and pathologists must be established to guide, assess, validate, and interpret the results of slide analysis from the perspective of responsibility and accountability to patients (45). Healthcare professional oversight is crucial for responsible decision-making and preventing harm, particularly in oncology.

## 5 Informed consent transformation in the era of digital pathology

A patient-centered approach incorporates DP tools in the laboratory of pathology transforms patient-physician relations and stimulates conversation about using AI-based algorithms for both clinicians and patients leading to fully informed decision-making (7). The use of AI-based tools and their impact on patients' health, should be disclosed before decision-making concerning diagnostics and treatment. The question arises as to how much data pathologists should disclose about AI-powered tools during the diagnostic process and whether patients should be given the choice of using traditional or AHI-based pathology.

Three aspects of informed consent are raised using AI-based technologies: the patient's right to know about using AI tools for further decision-making, privacy concerns and legal protection of patients. In general, the patient's right to accept or reject medical diagnostics and treatment correlates with the duty of physicians to provide information. This remains true regarding the use of AI-powered tools for diagnostics and treatment options recommendations. Patients must understand of the novel approaches used and determine their alignment with each patient's values. Ploug argues that patients should be provided with all of the information needed for decision making in terms of how the AI systems use the data about the patient and their histological slides, the potential biases of the AI-based system, how the labor is distributed between AI and health care professionals, and what the performance of AI-tool itself and in AI-Human team approach.

AI can provide deep insights into patients’ data, which may exacerbate privacy concerns (48, 49). Privacy possesses binary ethical meaning: the obligation to uphold and as a right to be protected (10). Application of DP power for the complex analysis of medical, histological, and molecular features allows for stratification of patients according to their risks on various cancer predispositions, predicts the probability of various mutations affecting patients' prognosis and also support health care professionals in defining the appropriate management options for every patient (46). However, the flip side of the issue is patients' data access and reliability. Providing AI tools access to personal data can provoke lead to privacy breaches and undesirable use of sensitive data. Individuals have a right to privacy and protection against harm. This implies a right to contest the use of health and other personal data during AI-supported diagnostics (50). This requires patients to be informed about the types of personal data used in AI diagnostics (for instance clinical data, laboratory tests, scans, WSI, etc.).

On the other hand, health data can be of different quality—some information can be outdated, one-sided, incomplete or erroneous (51). In such cases, the use of AI-based diagnostics based on the use of inaccurate personal health data can be inaccurate and harmful. This raises additional concerns about data sources and quality and reliability. Thus, as data sensitivity and quality depend on the source of the information, patients should be provided with the opportunity to contest the use of personal health data in AI-supported diagnostics with regard to data sources and types. Alternatively, patients could be provided with the opportunity to check and verify the data before they are retrieved to AI-based tools for diagnostics and prediction.

Providing patients with the choice of using AI-supported diagnostics rather than traditional pathology dictates the duties of healthcare institutions and laboratories to purchase and implement such systems in practice to enable patients access to innovative technologies and increase the quality of diagnostic services provided. Besides primary and regular staff training is essential for responsible implementation and use of emerging AI-based technologies. Both developers and users of AI-based systems should provide all the information about the logic, benefits and limitations if a patient raises concerns about the AI advice. Another ethical obligation of healthcare providers is to train the employees to provide information about AI systems to patients for a better understanding of AI-supported data analysis and decisions. Thus, the informed consent process should incorporate the disclosure of using DP tools for diagnostics and treatment options advice, the benefits and limitations of technology, considerations of data sources used as well as data protective measures. The next element of the informed consent process is cognition and understanding of information relying on the patient`s competence and comprehension but also depending on the relevant information provided. Standardized and clear disclosure of information in plain language enhances understanding of the technologies used (9). Besides patient education and public disclosure of the technological advances empower all stakeholders and improve public acceptance of the innovative technologies.

Assessment of various stakeholders' perspectives on using AI-based tools in medical practice highlights the importance of human involvement in the AI system's decisions and strategies for guarding against errors (45). AI can be involved in the diagnostic process differently. Some systems are used for initial screening prior to diagnosis, and others are directly involved in the diagnostic process, providing the preliminary report that must be validated by pathologists. The distribution of duties between AI and pathologists can improve the quality of the diagnostics and the whole performance of AI-Human teams while at the same time providing healthcare professionals and opportunity to make their own diagnostic decisions, articulating the responsibility of pathologists for conclusions made. Such an approach is essential to maintain patient trust and confidence in partially automated solutions in DP. Thus, respecting an individual's right to fully informed consent, patients should be notified about the use of AI, the type of data utilized and their sources, potential biases of the AI-based system related to training sets and models, diagnostic performance of the tool itself and in complex with health care professional, variables used for AI decision making and possible alternative information must also be provided about the proportion of AI and pathologists' labor, to clarify the role of AI and humans in making a decision and defining the responsibility for pathology report.

Patient choice between digital and traditional pathology raises legal and ethical issues. Patients have rights to bodily integrity and autonomy, but samples processing procedures and the use of various diagnostic tools rely on professional standards, so healthcare providers have the right to apply the most appropriate tests for professional decision-making (45). Several questions need to be answered to know how to best integrate AI including the question of whether AHI is ethically and legally distinctive from traditional pathology determinations in terms of risks/benefits ratio, that patients should have various options of choice on the degree automation applied in their care. Even while the use of AI continues to develop patients must be informed about the use of AI-based diagnostic tools that may be a basis for further treatment decisions. In full appreciation of shared decision-making patients should have a right to refuse AI-guided treatment recommendations if patients distrust AI technologies (45). More information about the use of AHI solutions and their benefits should be provided to patients and the public in general.

## 6 Conclusion

The application of DP provides multiple benefits for both healthcare professionals and patients. The AHI approach empowers pathologists to deliver accurate diagnoses and assess predictive biomarkers for further personalized treatment of cancer patients and improving their outcomes. However, the ethical implementation of DP requires revising physician-patient relationships. Such transformation dictates the duty of healthcare institutions and laboratories to provide the relevant information about AHI-based tools to cancer patients and the community for building transparency, understanding and trust, respecting patients' autonomy, and empowering informed decision-making in oncology.
